# 3-(2,4,6-Trimethyl­benzo­yl)-2-naphthoic acid

**DOI:** 10.1107/S1600536810040183

**Published:** 2010-10-13

**Authors:** S. Thenmozhi, J. ArulClement, A. K. MohanaKrishnan, A. SubbiahPandi

**Affiliations:** a Department of Physics, Presidency College (Autonomous), Chennai 600 005, India; b Department of Organic Chemistry, University of Madras, Guindy Campus, Chennai 600 025, India

## Abstract

The asymmetric unit of the title compound, C_21_H_18_O_3_, contains two crystallographically independent mol­ecules. The two mol­ecules are linked into cyclic centrosymmetric dimers *R*_2_^2^(8) by O—H⋯O hydrogen bonds. The dihedral angles between the naphthalene ring system and the benzene ring are 87.0 (8) and 84.4 (2)° in the two mol­ecules. The crystal packing is stabilized by O—H⋯O, C—H⋯π and π–π inter­actions [centroid–centroid distance = 3.664 (11) Å]. In one mol­ecule, the mesityl ring is disordered over two positions [occupancy ratio 0.690 (3):0.690 (3)].

## Related literature

For related structures, see: Ravishankar *et al.* (2005 ▶). For information on crystal engineering, see: Desiraju (2003 ▶); Almarsson & Zaworotko (2004 ▶).
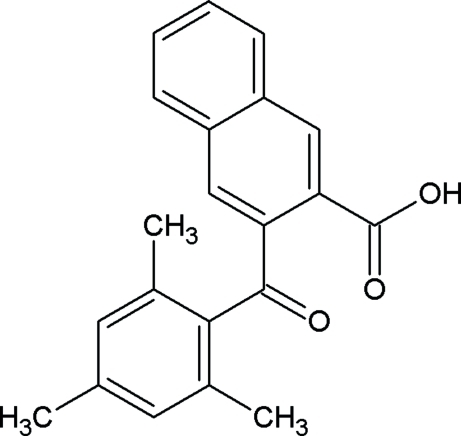


## Experimental

### 

#### Crystal data

C_21_H_18_O_3_
*M* *_r_* = 318.35
Triclinic, 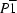

*a* = 10.5233 (3) Å
*b* = 12.7712 (3) Å
*c* = 13.0588 (3) Å
α = 93.102 (2)°
β = 101.609 (2)°
γ = 99.219 (2)°
*V* = 1690.15 (7) Å^3^
*Z* = 4
Mo *K*α radiation
μ = 0.08 mm^−1^
*T* = 293 K
0.30 × 0.20 × 0.20 mm

#### Data collection

Bruker APEXII CCD area detector diffractometer
Absorption correction: multi-scan (*SADABS*; Sheldrick, 1996 ▶) *T*_min_ = 0.976, *T*_max_ = 0.984
39093 measured reflections
8355 independent reflections
5564 reflections with *I* > 2σ(*I*)
*R*_int_ = 0.028

#### Refinement

*R*[*F* ^2^ > 2σ(*F* ^2^)] = 0.049
*wR*(*F* ^2^) = 0.167
*S* = 1.04
8355 reflections
472 parameters
1 restraint
H-atom parameters constrained
Δρ_max_ = 0.28 e Å^−3^
Δρ_min_ = −0.23 e Å^−3^

### 

Data collection: *APEX2* (Bruker, 2004 ▶); cell refinement: *SAINT* (Bruker, 2004 ▶); data reduction: *SAINT*; program(s) used to solve structure: *SHELXS97* (Sheldrick, 2008 ▶); program(s) used to refine structure: *SHELXL97* (Sheldrick, 2008 ▶); molecular graphics: *ORTEP-3* (Farrugia, 1997 ▶); software used to prepare material for publication: *SHELXL97* and *PLATON* (Spek, 2009 ▶).

## Supplementary Material

Crystal structure: contains datablocks global, I. DOI: 10.1107/S1600536810040183/bt5351sup1.cif

Structure factors: contains datablocks I. DOI: 10.1107/S1600536810040183/bt5351Isup2.hkl

Additional supplementary materials:  crystallographic information; 3D view; checkCIF report

## Figures and Tables

**Table 1:** Hydrogen-bond geometry (Å, °) *Cg*1 and *Cg*2 are the centroids of the C22–C27 and C26–C31rings, respectively.

*D*—H⋯*A*	*D*—H	H⋯*A*	*D*⋯*A*	*D*—H⋯*A*
O2—H2*A*⋯O3^i^	0.82	1.81	2.6191 (17)	171
O6—H6*A*⋯O5^ii^	0.82	1.86	2.6709 (18)	170
C21—H21*A*⋯*Cg*7^iii^	0.96	2.85	3.475 (2)	124
C31—H31⋯*Cg*7	0.93	2.71	3.548 (2)	150
C42—H42*B*⋯*Cg*6^iv^	0.96	2.86	3.620 (6)	137

Symmetry codes: (i) 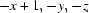; (ii) 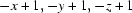; (iii) 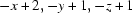; (iv) 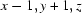.

**Fig. 1.**
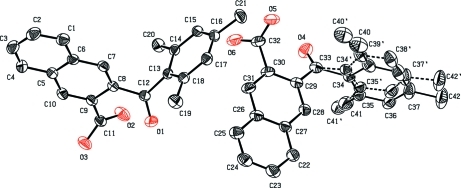
The structure of showing the atom-numbering scheme. Displacement ellipsoids are drawn at the 30% probability level. For the sake of clarity the H atoms have been omitted.

**Fig. 2.**
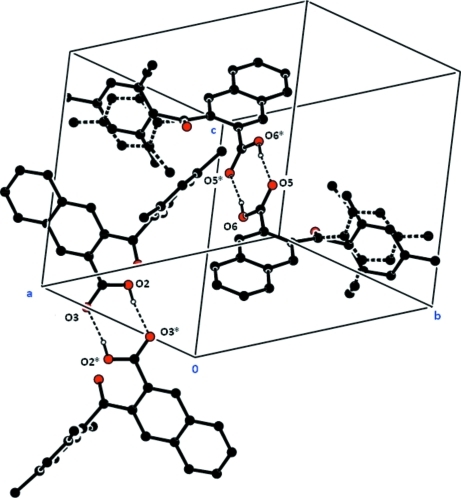
The crystal structure showing the formation of the centrosymmetric R22(8) dimer for both the molecules. For the sake of clarity, the H atoms not involved in the motif have been omitted. The atoms marked with an asterisk (*) are at the symmetry positions (1 - x, 1 - y, 1 - z) and (1 - x, -y, -z). The dashed lines indicate hydrogen bonds.

## supplementary crystallographic information

### Comment

Crystal engineering of organic molecules has been exploited in organic materials and in active pharmaceutical ingredients (Desiraju, 2003; Almarsson & Zaworotko, 2004). Supra molecular synthons provide an effective stratedy for synthesizing specific organic supramolecularsolids. 3-Hydroxy-2-naphthoicacid, also known as -oxy-naphthoic acid (BONA, BONS), is produced industrially in a 1000 ton scale.

Fig. 1 shows the asymmetric unit consisting of two molecules of the title compound. The two crystallographically independent molecules have the same geometrical parameters within the precision of the experiments.   The geometric parameters of the title molecule agree well with those reported for a similar structure (Ravishankar *et al.*, 2005). The naphthalene ring systems makes dihedral angles of 87.0 (8)° and 84.4 (16)° with the mesityl ring system.

In addition to van der Waals interaction, the crystal packing is stabilized by C–H..O, C–H···π and (Table. 1) hydrogen bonds as well as by π–π electron interactions. In the crystal structure one of the molecules at (*x*, *y*, *z*) and (1 - *x*, -*y*, -*z*) are linked by O2–H2A···O3 hydrogen bonds into a cyclic centrosymmetric *R*_2_^2^(8) dimer and also for other molecule at (*x*, *y*, *z*) and (1 - *x*, 1 - *y*, 1 - *z*) are linked by O6–H6A···O5 hydrogen bonds into a cyclic centrosymmetric *R*_2_^2^(8) dimer (Fig. 2). The π–π electron interactions between the rings *Cg*1···*Cg*2 at 1/2 - *x*,1/2 + *y*,1/2 - *z* with the centroid-centroid distance equal to 3.664 (11) Å, is observed in the crystal structure [*Cg*1 and *Cg*2 are the centeroid of the rings C22–C27 and C26–C31].

### Experimental

The ethyl 4-mesityl-4-oxobutanote (3.0 g, 12.09 mmol) and phthalaldehyde (1.62 g, 12.09 mmol) were dissolved in hot ethanol (60 ml) to the reaction mixture tertiary-Butane oxide (3.38 g, 30.24 mmol) was slowly added and the reaction mixture was stirred for 10 h at room temperature. Then it was poured in to ice-water (200 ml) and extracted with DCM (40 ml). The aqueous layer was acidified using HCl (PH=2–3) and it was stirred for 0.5 h at room temperature. The solid obtained was filtered and washed with methanol(40 ml) to afford 3-(2,4,6 trimethyl benzoyl)-2–2 napthoic acid. Single crystals suitable for X-ray diffraction were obtained by slow evaporation of a solution of the title compound in chloroform at room temperature.

### Refinement

All H atoms were fixed geometrically and allowed to ride on their parent C atoms, with C—H distances fixed in the range 0.93–0.97 Å with *U*_iso_(H) = 1.5*U*_eq_(C) for methyl H 1.2*U*_eq_(C) for other H atoms.

### Crystal data

**Table:**

C_21_H_18_O_3_	*Z* = 4
*M**_r_* = 318.35	*F*(000) = 672
Triclinic, *P*1	*D*_x_ = 1.251 Mg m^−^^3^
Hall symbol: -P 1	Mo *K*α radiation, λ = 0.71073 Å
*a* = 10.5233 (3) Å	Cell parameters from 8355 reflections
*b* = 12.7712 (3) Å	θ = 1.6–28.3°
*c* = 13.0588 (3) Å	µ = 0.08 mm^−^^1^
α = 93.102 (2)°	*T* = 293 K
β = 101.609 (2)°	Block, white crystalline
γ = 99.219 (2)°	0.30 × 0.20 × 0.20 mm
*V* = 1690.15 (7) Å^3^	

### Data collection

**Table:**

Bruker APEXII CCD area detector diffractometer	8355 independent reflections
Radiation source: fine-focus sealed tube	5564 reflections with *I* > 2σ(*I*)
graphite	*R*_int_ = 0.028
ω and φ scans	θ_max_ = 28.3°, θ_min_ = 1.6°
Absorption correction: multi-scan (*SADABS*; Sheldrick, 1996)	*h* = −14→14
*T*_min_ = 0.976, *T*_max_ = 0.984	*k* = −17→17
39093 measured reflections	*l* = −17→17

### Refinement

**Table:**

Refinement on *F*^2^	Secondary atom site location: difference Fourier map
Least-squares matrix: full	Hydrogen site location: inferred from neighbouring sites
*R*[*F*^2^ > 2σ(*F*^2^)] = 0.049	H-atom parameters constrained
*wR*(*F*^2^) = 0.167	*w* = 1/[σ^2^(*F*_o_^2^) + (0.0838*P*)^2^ + 0.2658*P*] where *P* = (*F*_o_^2^ + 2*F*_c_^2^)/3
*S* = 1.04	(Δ/σ)_max_ = 0.002
8355 reflections	Δρ_max_ = 0.28 e Å^−^^3^
472 parameters	Δρ_min_ = −0.23 e Å^−^^3^
1 restraint	Extinction correction: *SHELXL97* (Sheldrick, 2008), Fc^*^=kFc[1+0.001xFc^2^λ^3^/sin(2θ)]^-1/4^
Primary atom site location: structure-invariant direct methods	Extinction coefficient: 0.0058 (16)

### Special details

**Table:**

Geometry. All e.s.d.'s (except the e.s.d. in the dihedral angle between two l.s. planes) are estimated using the full covariance matrix. The cell e.s.d.'s are taken into account individually in the estimation of e.s.d.'s in distances, angles and torsion angles; correlations between e.s.d.'s in cell parameters are only used when they are defined by crystal symmetry. An approximate (isotropic) treatment of cell e.s.d.'s is used for estimating e.s.d.'s involving l.s. planes.
Refinement. Refinement of *F*^2^ against ALL reflections. The weighted *R*-factor *wR* and goodness of fit *S* are based on *F*^2^, conventional *R*-factors *R* are based on *F*, with *F* set to zero for negative *F*^2^. The threshold expression of *F*^2^ > σ(*F*^2^) is used only for calculating *R*-factors(gt) *etc*. and is not relevant to the choice of reflections for refinement. *R*-factors based on *F*^2^ are statistically about twice as large as those based on *F*, and *R*- factors based on ALL data will be even larger.

### Fractional atomic coordinates and isotropic or equivalent isotropic displacement parameters (Å^2^)

**Table:**

	*x*	*y*	*z*	*U*_iso_*/*U*_eq_	Occ. (<1)
C1	1.11744 (17)	0.05037 (16)	0.42909 (14)	0.0597 (4)	
H1	1.1613	0.1155	0.4641	0.072*	
C2	1.1637 (2)	−0.04010 (19)	0.45394 (16)	0.0707 (5)	
H2	1.2395	−0.0363	0.5059	0.085*	
C3	1.0997 (2)	−0.13863 (19)	0.40314 (17)	0.0728 (6)	
H3	1.1329	−0.1999	0.4214	0.087*	
C4	0.9894 (2)	−0.14580 (15)	0.32728 (15)	0.0658 (5)	
H4	0.9467	−0.2121	0.2942	0.079*	
C5	0.93850 (17)	−0.05327 (13)	0.29797 (13)	0.0506 (4)	
C6	1.00277 (15)	0.04601 (13)	0.35011 (12)	0.0471 (4)	
C7	0.95130 (15)	0.13796 (12)	0.32070 (12)	0.0450 (3)	
H7	0.9925	0.2035	0.3562	0.054*	
C8	0.84305 (14)	0.13400 (11)	0.24178 (11)	0.0405 (3)	
C9	0.77928 (15)	0.03304 (11)	0.18832 (12)	0.0438 (3)	
C10	0.82655 (17)	−0.05676 (13)	0.21683 (13)	0.0520 (4)	
H10	0.7838	−0.1222	0.1818	0.062*	
C11	0.65636 (15)	0.02060 (12)	0.10617 (12)	0.0460 (4)	
C12	0.80778 (14)	0.23579 (12)	0.20428 (12)	0.0434 (3)	
C13	0.82973 (14)	0.33111 (11)	0.28178 (11)	0.0402 (3)	
C14	0.75382 (14)	0.33287 (11)	0.35780 (11)	0.0416 (3)	
C15	0.77141 (15)	0.42539 (12)	0.42389 (12)	0.0459 (3)	
H15	0.7229	0.4267	0.4760	0.055*	
C16	0.85886 (16)	0.51545 (12)	0.41436 (12)	0.0484 (4)	
C17	0.93509 (16)	0.51054 (12)	0.34061 (13)	0.0508 (4)	
H17	0.9962	0.5701	0.3353	0.061*	
C18	0.92364 (15)	0.41989 (12)	0.27422 (12)	0.0456 (3)	
C19	1.01500 (19)	0.41711 (16)	0.19955 (16)	0.0669 (5)	
H19A	0.9722	0.4334	0.1317	0.100*	
H19B	1.0375	0.3474	0.1940	0.100*	
H19C	1.0937	0.4687	0.2253	0.100*	
C20	0.64975 (18)	0.24030 (14)	0.36671 (15)	0.0589 (4)	
H20A	0.6184	0.2523	0.4298	0.088*	
H20B	0.6866	0.1762	0.3690	0.088*	
H20C	0.5778	0.2334	0.3071	0.088*	
C21	0.8693 (2)	0.61697 (14)	0.48227 (16)	0.0655 (5)	
H21A	0.9598	0.6514	0.5020	0.098*	
H21B	0.8367	0.6007	0.5442	0.098*	
H21C	0.8180	0.6635	0.4439	0.098*	
C22	0.5784 (2)	0.69010 (15)	−0.02850 (15)	0.0689 (5)	
H22	0.5372	0.7429	−0.0600	0.083*	
C23	0.6618 (2)	0.64349 (16)	−0.07632 (17)	0.0733 (6)	
H23	0.6775	0.6654	−0.1401	0.088*	
C24	0.7238 (2)	0.56368 (17)	−0.03095 (16)	0.0688 (5)	
H24	0.7807	0.5331	−0.0645	0.083*	
C25	0.70201 (18)	0.53005 (17)	0.06189 (15)	0.0650 (5)	
H25	0.7434	0.4763	0.0913	0.078*	
C26	0.61658 (16)	0.57651 (15)	0.11395 (12)	0.0534 (4)	
C27	0.55480 (17)	0.65819 (13)	0.06865 (13)	0.0514 (4)	
C28	0.46686 (18)	0.70208 (13)	0.12034 (13)	0.0530 (4)	
H28	0.4259	0.7560	0.0910	0.064*	
C29	0.44055 (16)	0.66714 (13)	0.21255 (12)	0.0481 (4)	
C30	0.50410 (16)	0.58553 (15)	0.25766 (12)	0.0528 (4)	
C31	0.58855 (17)	0.54186 (17)	0.20916 (13)	0.0605 (5)	
H31	0.6288	0.4879	0.2394	0.073*	
C32	0.49066 (17)	0.55014 (17)	0.36282 (13)	0.0574 (4)	
C33	0.33412 (16)	0.70412 (13)	0.25617 (13)	0.0509 (4)	
C34	0.2862 (4)	0.8075 (5)	0.2189 (3)	0.0450 (10)	0.690 (3)
C35	0.1698 (4)	0.8031 (4)	0.1440 (3)	0.0511 (9)	0.690 (3)
C36	0.1307 (4)	0.8974 (3)	0.1156 (3)	0.0674 (9)	0.690 (3)
H36	0.0542	0.8947	0.0646	0.081*	0.690 (3)
C37	0.2009 (5)	0.9959 (3)	0.1600 (4)	0.0768 (12)	0.690 (3)
C38	0.3163 (4)	0.9983 (3)	0.2342 (4)	0.0763 (19)	0.690 (3)
H38	0.3651	1.0637	0.2649	0.092*	0.690 (3)
C39	0.3606 (4)	0.9055 (3)	0.2635 (3)	0.0610 (9)	0.690 (3)
C40	0.4859 (4)	0.9130 (4)	0.3451 (4)	0.0928 (13)	0.690 (3)
H40A	0.4699	0.8687	0.3998	0.139*	0.690 (3)
H40B	0.5154	0.9856	0.3743	0.139*	0.690 (3)
H40C	0.5523	0.8897	0.3132	0.139*	0.690 (3)
C41	0.0880 (6)	0.6981 (4)	0.0961 (4)	0.0738 (11)	0.690 (3)
H41A	0.0451	0.6647	0.1473	0.111*	0.690 (3)
H41B	0.1437	0.6529	0.0737	0.111*	0.690 (3)
H41C	0.0227	0.7094	0.0369	0.111*	0.690 (3)
C42	0.1530 (6)	1.0968 (3)	0.1284 (5)	0.1149 (18)	0.690 (3)
H42A	0.1990	1.1550	0.1786	0.172*	0.690 (3)
H42B	0.0602	1.0887	0.1261	0.172*	0.690 (3)
H42C	0.1694	1.1109	0.0603	0.172*	0.690 (3)
C34'	0.3195 (13)	0.8079 (13)	0.2462 (9)	0.0450 (10)	0.310 (3)
C35'	0.2097 (13)	0.8196 (10)	0.1747 (8)	0.0511 (9)	0.310 (3)
C36'	0.1871 (9)	0.9259 (9)	0.1584 (7)	0.0674 (9)	0.310 (3)
H36'	0.1144	0.9378	0.1096	0.081*	0.310 (3)
C37'	0.2724 (17)	1.0074 (13)	0.2146 (16)	0.0768 (12)	0.310 (3)
C38'	0.3806 (10)	0.9930 (8)	0.2864 (9)	0.0763 (19)	0.310 (3)
H38'	0.4371	1.0521	0.3244	0.092*	0.310 (3)
C39'	0.4073 (10)	0.8927 (8)	0.3034 (8)	0.0610 (9)	0.310 (3)
C40'	0.5246 (11)	0.8720 (9)	0.3837 (8)	0.0928 (13)	0.310 (3)
H40D	0.6034	0.8896	0.3573	0.139*	0.310 (3)
H40E	0.5120	0.7982	0.3967	0.139*	0.310 (3)
H40F	0.5324	0.9152	0.4480	0.139*	0.310 (3)
C41'	0.1120 (16)	0.7329 (10)	0.1118 (12)	0.0738 (11)	0.310 (3)
H41D	0.1455	0.7075	0.0537	0.111*	0.310 (3)
H41E	0.0318	0.7587	0.0859	0.111*	0.310 (3)
H41F	0.0949	0.6757	0.1547	0.111*	0.310 (3)
C42'	0.2527 (13)	1.1240 (8)	0.1984 (11)	0.1149 (18)	0.310 (3)
H42D	0.2719	1.1424	0.1321	0.172*	0.310 (3)
H42E	0.3110	1.1713	0.2539	0.172*	0.310 (3)
H42F	0.1632	1.1301	0.1991	0.172*	0.310 (3)
O1	0.76841 (14)	0.24315 (10)	0.11138 (9)	0.0673 (4)	
O2	0.56779 (12)	0.07128 (11)	0.12213 (10)	0.0708 (4)	
H2A	0.5041	0.0568	0.0728	0.085*	
O3	0.64372 (13)	−0.04411 (10)	0.02849 (10)	0.0696 (4)	
O4	0.27640 (14)	0.64882 (12)	0.31134 (11)	0.0734 (4)	
O5	0.52720 (15)	0.61471 (12)	0.44170 (9)	0.0739 (4)	
O6	0.44929 (14)	0.45080 (13)	0.36565 (10)	0.0740 (4)	
H6A	0.4530	0.4375	0.4268	0.089*	

### Atomic displacement parameters (Å^2^)

**Table:**

	*U*^11^	*U*^22^	*U*^33^	*U*^12^	*U*^13^	*U*^23^
C1	0.0540 (10)	0.0772 (12)	0.0505 (10)	0.0226 (9)	0.0080 (8)	0.0064 (8)
C2	0.0632 (11)	0.0998 (16)	0.0595 (11)	0.0382 (11)	0.0138 (9)	0.0235 (11)
C3	0.0880 (14)	0.0837 (14)	0.0660 (12)	0.0502 (12)	0.0285 (11)	0.0262 (11)
C4	0.0913 (14)	0.0579 (11)	0.0590 (11)	0.0336 (10)	0.0239 (10)	0.0111 (8)
C5	0.0628 (10)	0.0517 (9)	0.0438 (8)	0.0211 (7)	0.0174 (7)	0.0049 (7)
C6	0.0494 (8)	0.0573 (9)	0.0391 (8)	0.0185 (7)	0.0128 (6)	0.0033 (7)
C7	0.0471 (8)	0.0458 (8)	0.0402 (8)	0.0084 (6)	0.0068 (6)	−0.0046 (6)
C8	0.0417 (7)	0.0410 (7)	0.0384 (7)	0.0070 (6)	0.0089 (6)	−0.0026 (6)
C9	0.0478 (8)	0.0417 (8)	0.0404 (8)	0.0057 (6)	0.0092 (6)	−0.0028 (6)
C10	0.0660 (10)	0.0399 (8)	0.0495 (9)	0.0079 (7)	0.0138 (8)	−0.0032 (6)
C11	0.0491 (8)	0.0415 (8)	0.0438 (8)	0.0017 (6)	0.0084 (7)	−0.0045 (6)
C12	0.0440 (8)	0.0432 (8)	0.0398 (8)	0.0055 (6)	0.0042 (6)	−0.0003 (6)
C13	0.0438 (7)	0.0360 (7)	0.0387 (7)	0.0087 (6)	0.0028 (6)	0.0023 (6)
C14	0.0416 (7)	0.0407 (7)	0.0416 (8)	0.0102 (6)	0.0038 (6)	0.0057 (6)
C15	0.0471 (8)	0.0509 (8)	0.0405 (8)	0.0167 (7)	0.0051 (6)	0.0018 (6)
C16	0.0515 (9)	0.0427 (8)	0.0456 (8)	0.0130 (7)	−0.0045 (7)	−0.0025 (6)
C17	0.0535 (9)	0.0391 (8)	0.0538 (9)	0.0016 (7)	0.0029 (7)	0.0027 (7)
C18	0.0472 (8)	0.0428 (8)	0.0444 (8)	0.0048 (6)	0.0061 (6)	0.0041 (6)
C19	0.0667 (11)	0.0641 (11)	0.0706 (12)	−0.0029 (9)	0.0290 (10)	0.0018 (9)
C20	0.0577 (10)	0.0529 (9)	0.0676 (11)	0.0035 (8)	0.0224 (8)	0.0033 (8)
C21	0.0725 (12)	0.0513 (10)	0.0656 (11)	0.0171 (9)	−0.0019 (9)	−0.0133 (8)
C22	0.1051 (15)	0.0509 (10)	0.0631 (11)	0.0133 (10)	0.0451 (11)	0.0123 (8)
C23	0.1048 (16)	0.0604 (11)	0.0667 (12)	0.0052 (11)	0.0533 (12)	0.0072 (9)
C24	0.0683 (12)	0.0783 (13)	0.0674 (12)	0.0100 (10)	0.0366 (10)	−0.0018 (10)
C25	0.0556 (10)	0.0895 (14)	0.0569 (10)	0.0254 (10)	0.0188 (8)	0.0057 (9)
C26	0.0474 (8)	0.0726 (11)	0.0416 (8)	0.0128 (8)	0.0118 (7)	0.0028 (7)
C27	0.0626 (10)	0.0477 (8)	0.0463 (9)	0.0051 (7)	0.0219 (7)	0.0011 (7)
C28	0.0663 (10)	0.0488 (9)	0.0490 (9)	0.0123 (8)	0.0224 (8)	0.0060 (7)
C29	0.0503 (8)	0.0553 (9)	0.0393 (8)	0.0090 (7)	0.0122 (6)	0.0007 (7)
C30	0.0497 (9)	0.0768 (11)	0.0342 (8)	0.0175 (8)	0.0087 (6)	0.0075 (7)
C31	0.0569 (10)	0.0901 (13)	0.0409 (9)	0.0309 (9)	0.0092 (7)	0.0142 (8)
C32	0.0518 (9)	0.0877 (14)	0.0385 (9)	0.0251 (9)	0.0107 (7)	0.0121 (9)
C33	0.0531 (9)	0.0578 (9)	0.0438 (8)	0.0090 (7)	0.0159 (7)	0.0026 (7)
C34	0.045 (3)	0.0505 (10)	0.043 (3)	0.010 (2)	0.015 (2)	0.003 (2)
C35	0.055 (3)	0.059 (2)	0.045 (2)	0.017 (2)	0.0159 (16)	0.0128 (19)
C36	0.069 (2)	0.080 (2)	0.068 (2)	0.0308 (19)	0.0286 (16)	0.0298 (19)
C37	0.096 (3)	0.0581 (17)	0.106 (3)	0.034 (2)	0.066 (2)	0.037 (2)
C38	0.086 (4)	0.0493 (18)	0.103 (5)	0.002 (3)	0.051 (3)	0.002 (3)
C39	0.060 (2)	0.0567 (15)	0.069 (3)	0.0038 (16)	0.0278 (17)	−0.0039 (17)
C40	0.080 (3)	0.096 (3)	0.085 (3)	−0.011 (2)	0.005 (2)	−0.014 (2)
C41	0.078 (3)	0.069 (3)	0.063 (2)	0.004 (2)	−0.0038 (18)	0.002 (2)
C42	0.150 (4)	0.074 (2)	0.168 (5)	0.057 (3)	0.102 (4)	0.063 (3)
C34'	0.045 (3)	0.0505 (10)	0.043 (3)	0.010 (2)	0.015 (2)	0.003 (2)
C35'	0.055 (3)	0.059 (2)	0.045 (2)	0.017 (2)	0.0159 (16)	0.0128 (19)
C36'	0.069 (2)	0.080 (2)	0.068 (2)	0.0308 (19)	0.0286 (16)	0.0298 (19)
C37'	0.096 (3)	0.0581 (17)	0.106 (3)	0.034 (2)	0.066 (2)	0.037 (2)
C38'	0.086 (4)	0.0493 (18)	0.103 (5)	0.002 (3)	0.051 (3)	0.002 (3)
C39'	0.060 (2)	0.0567 (15)	0.069 (3)	0.0038 (16)	0.0278 (17)	−0.0039 (17)
C40'	0.080 (3)	0.096 (3)	0.085 (3)	−0.011 (2)	0.005 (2)	−0.014 (2)
C41'	0.078 (3)	0.069 (3)	0.063 (2)	0.004 (2)	−0.0038 (18)	0.002 (2)
C42'	0.150 (4)	0.074 (2)	0.168 (5)	0.057 (3)	0.102 (4)	0.063 (3)
O1	0.0956 (10)	0.0584 (7)	0.0407 (6)	0.0145 (7)	−0.0028 (6)	0.0014 (5)
O2	0.0511 (7)	0.0850 (9)	0.0661 (8)	0.0145 (6)	−0.0042 (6)	−0.0276 (7)
O3	0.0726 (8)	0.0690 (8)	0.0576 (7)	0.0179 (6)	−0.0046 (6)	−0.0244 (6)
O4	0.0734 (9)	0.0858 (9)	0.0798 (9)	0.0290 (7)	0.0405 (7)	0.0359 (7)
O5	0.0908 (10)	0.0980 (10)	0.0374 (6)	0.0296 (8)	0.0136 (6)	0.0064 (7)
O6	0.0879 (10)	0.0938 (11)	0.0448 (7)	0.0203 (8)	0.0176 (6)	0.0200 (7)

### Geometric parameters (Å, °)

**Table:**

C1—C2	1.354 (3)	C28—C29	1.371 (2)
C1—C6	1.412 (2)	C28—H28	0.9300
C1—H1	0.9300	C29—C30	1.418 (2)
C2—C3	1.391 (3)	C29—C33	1.483 (2)
C2—H2	0.9300	C30—C31	1.360 (2)
C3—C4	1.353 (3)	C30—C32	1.495 (2)
C3—H3	0.9300	C31—H31	0.9300
C4—C5	1.414 (2)	C32—O5	1.239 (2)
C4—H4	0.9300	C32—O6	1.279 (2)
C5—C6	1.405 (2)	C33—O4	1.2167 (19)
C5—C10	1.410 (2)	C33—C34'	1.369 (17)
C6—C7	1.410 (2)	C33—C34	1.555 (6)
C7—C8	1.366 (2)	C34—C39	1.393 (7)
C7—H7	0.9300	C34—C35	1.397 (4)
C8—C9	1.4272 (19)	C35—C36	1.379 (6)
C8—C12	1.489 (2)	C35—C41	1.502 (5)
C9—C10	1.362 (2)	C36—C37	1.384 (6)
C9—C11	1.486 (2)	C36—H36	0.9300
C10—H10	0.9300	C37—C38	1.388 (6)
C11—O3	1.2447 (18)	C37—C42	1.506 (5)
C11—O2	1.2602 (19)	C38—C39	1.387 (6)
C12—O1	1.2144 (18)	C38—H38	0.9300
C12—C13	1.497 (2)	C39—C40	1.505 (5)
C13—C14	1.395 (2)	C40—H40A	0.9600
C13—C18	1.402 (2)	C40—H40B	0.9600
C14—C15	1.389 (2)	C40—H40C	0.9600
C14—C20	1.504 (2)	C41—H41A	0.9600
C15—C16	1.381 (2)	C41—H41B	0.9600
C15—H15	0.9300	C41—H41C	0.9600
C16—C17	1.377 (2)	C42—H42A	0.9600
C16—C21	1.507 (2)	C42—H42B	0.9600
C17—C18	1.382 (2)	C42—H42C	0.9600
C17—H17	0.9300	C34'—C35'	1.366 (11)
C18—C19	1.504 (2)	C34'—C39'	1.378 (17)
C19—H19A	0.9600	C35'—C36'	1.435 (17)
C19—H19B	0.9600	C35'—C41'	1.468 (12)
C19—H19C	0.9600	C36'—C37'	1.33 (2)
C20—H20A	0.9600	C36'—H36'	0.9300
C20—H20B	0.9600	C37'—C38'	1.37 (2)
C20—H20C	0.9600	C37'—C42'	1.556 (17)
C21—H21A	0.9600	C38'—C39'	1.376 (15)
C21—H21B	0.9600	C38'—H38'	0.9300
C21—H21C	0.9600	C39'—C40'	1.519 (13)
C22—C23	1.365 (3)	C40'—H40D	0.9600
C22—C27	1.410 (2)	C40'—H40E	0.9600
C22—H22	0.9300	C40'—H40F	0.9600
C23—C24	1.392 (3)	C41'—H41D	0.9600
C23—H23	0.9300	C41'—H41E	0.9600
C24—C25	1.358 (3)	C41'—H41F	0.9600
C24—H24	0.9300	C42'—H42D	0.9600
C25—C26	1.413 (2)	C42'—H42E	0.9600
C25—H25	0.9300	C42'—H42F	0.9600
C26—C27	1.411 (2)	O2—H2A	0.8200
C26—C31	1.412 (2)	O6—H6A	0.8200
C27—C28	1.415 (2)		
			
C2—C1—C6	120.14 (19)	C27—C26—C31	118.78 (15)
C2—C1—H1	119.9	C27—C26—C25	119.32 (15)
C6—C1—H1	119.9	C31—C26—C25	121.89 (17)
C1—C2—C3	121.12 (19)	C22—C27—C26	118.96 (16)
C1—C2—H2	119.4	C22—C27—C28	122.30 (17)
C3—C2—H2	119.4	C26—C27—C28	118.68 (14)
C4—C3—C2	120.33 (18)	C29—C28—C27	121.69 (16)
C4—C3—H3	119.8	C29—C28—H28	119.2
C2—C3—H3	119.8	C27—C28—H28	119.2
C3—C4—C5	120.45 (19)	C28—C29—C30	119.05 (15)
C3—C4—H4	119.8	C28—C29—C33	119.87 (15)
C5—C4—H4	119.8	C30—C29—C33	120.58 (14)
C6—C5—C10	118.67 (15)	C31—C30—C29	120.28 (15)
C6—C5—C4	119.05 (16)	C31—C30—C32	117.20 (16)
C10—C5—C4	122.27 (16)	C29—C30—C32	122.34 (15)
C5—C6—C7	118.75 (14)	C30—C31—C26	121.51 (17)
C5—C6—C1	118.91 (15)	C30—C31—H31	119.2
C7—C6—C1	122.34 (15)	C26—C31—H31	119.2
C8—C7—C6	122.24 (14)	O5—C32—O6	123.88 (16)
C8—C7—H7	118.9	O5—C32—C30	119.81 (18)
C6—C7—H7	118.9	O6—C32—C30	116.14 (16)
C7—C8—C9	118.67 (14)	O4—C33—C34'	121.7 (6)
C7—C8—C12	118.74 (13)	O4—C33—C29	120.35 (16)
C9—C8—C12	121.97 (13)	C34'—C33—C29	117.1 (6)
C10—C9—C8	119.84 (14)	O4—C33—C34	120.5 (2)
C10—C9—C11	117.82 (13)	C34'—C33—C34	15.9 (5)
C8—C9—C11	122.22 (13)	C29—C33—C34	118.7 (2)
C9—C10—C5	121.81 (14)	C39—C34—C35	120.2 (5)
C9—C10—H10	119.1	C39—C34—C33	118.6 (2)
C5—C10—H10	119.1	C35—C34—C33	121.1 (4)
O3—C11—O2	123.77 (15)	C36—C35—C34	118.6 (4)
O3—C11—C9	117.98 (14)	C36—C35—C41	120.6 (4)
O2—C11—C9	118.11 (13)	C34—C35—C41	120.8 (5)
O1—C12—C8	120.52 (13)	C35—C36—C37	122.5 (3)
O1—C12—C13	120.25 (14)	C35—C36—H36	118.8
C8—C12—C13	119.11 (12)	C37—C36—H36	118.8
C14—C13—C18	120.57 (13)	C36—C37—C38	117.9 (3)
C14—C13—C12	120.25 (13)	C36—C37—C42	120.7 (4)
C18—C13—C12	119.13 (13)	C38—C37—C42	121.4 (5)
C15—C14—C13	118.44 (13)	C39—C38—C37	121.5 (4)
C15—C14—C20	119.32 (14)	C39—C38—H38	119.3
C13—C14—C20	122.17 (13)	C37—C38—H38	119.3
C16—C15—C14	121.85 (15)	C38—C39—C34	119.3 (4)
C16—C15—H15	119.1	C38—C39—C40	119.1 (4)
C14—C15—H15	119.1	C34—C39—C40	121.6 (3)
C17—C16—C15	118.43 (14)	C35'—C34'—C33	113.9 (12)
C17—C16—C21	120.86 (16)	C35'—C34'—C39'	123.1 (15)
C15—C16—C21	120.70 (16)	C33—C34'—C39'	123.0 (10)
C16—C17—C18	122.12 (14)	C34'—C35'—C36'	117.7 (12)
C16—C17—H17	118.9	C34'—C35'—C41'	125.9 (14)
C18—C17—H17	118.9	C36'—C35'—C41'	116.4 (12)
C17—C18—C13	118.48 (14)	C37'—C36'—C35'	118.6 (10)
C17—C18—C19	119.70 (14)	C37'—C36'—H36'	120.7
C13—C18—C19	121.77 (14)	C35'—C36'—H36'	120.7
C18—C19—H19A	109.5	C36'—C37'—C38'	122.3 (15)
C18—C19—H19B	109.5	C36'—C37'—C42'	120.3 (15)
H19A—C19—H19B	109.5	C38'—C37'—C42'	117.3 (16)
C18—C19—H19C	109.5	C37'—C38'—C39'	121.0 (13)
H19A—C19—H19C	109.5	C37'—C38'—H38'	119.5
H19B—C19—H19C	109.5	C39'—C38'—H38'	119.5
C14—C20—H20A	109.5	C38'—C39'—C34'	117.2 (10)
C14—C20—H20B	109.5	C38'—C39'—C40'	123.2 (9)
H20A—C20—H20B	109.5	C34'—C39'—C40'	119.5 (10)
C14—C20—H20C	109.5	C39'—C40'—H40D	109.5
H20A—C20—H20C	109.5	C39'—C40'—H40E	109.5
H20B—C20—H20C	109.5	H40D—C40'—H40E	109.5
C16—C21—H21A	109.5	C39'—C40'—H40F	109.5
C16—C21—H21B	109.5	H40D—C40'—H40F	109.5
H21A—C21—H21B	109.5	H40E—C40'—H40F	109.5
C16—C21—H21C	109.5	C35'—C41'—H41D	109.5
H21A—C21—H21C	109.5	C35'—C41'—H41E	109.5
H21B—C21—H21C	109.5	H41D—C41'—H41E	109.5
C23—C22—C27	120.01 (19)	C35'—C41'—H41F	109.5
C23—C22—H22	120.0	H41D—C41'—H41F	109.5
C27—C22—H22	120.0	H41E—C41'—H41F	109.5
C22—C23—C24	120.93 (17)	C37'—C42'—H42D	109.5
C22—C23—H23	119.5	C37'—C42'—H42E	109.5
C24—C23—H23	119.5	H42D—C42'—H42E	109.5
C25—C24—C23	120.62 (18)	C37'—C42'—H42F	109.5
C25—C24—H24	119.7	H42D—C42'—H42F	109.5
C23—C24—H24	119.7	H42E—C42'—H42F	109.5
C24—C25—C26	120.14 (19)	C11—O2—H2A	109.5
C24—C25—H25	119.9	C32—O6—H6A	109.5
C26—C25—H25	119.9		
			
C6—C1—C2—C3	−0.3 (3)	C27—C28—C29—C30	0.6 (2)
C1—C2—C3—C4	0.1 (3)	C27—C28—C29—C33	−171.35 (15)
C2—C3—C4—C5	0.6 (3)	C28—C29—C30—C31	−0.8 (3)
C3—C4—C5—C6	−1.2 (3)	C33—C29—C30—C31	171.02 (16)
C3—C4—C5—C10	177.72 (17)	C28—C29—C30—C32	174.13 (16)
C10—C5—C6—C7	1.2 (2)	C33—C29—C30—C32	−14.0 (2)
C4—C5—C6—C7	−179.81 (15)	C29—C30—C31—C26	0.6 (3)
C10—C5—C6—C1	−177.95 (15)	C32—C30—C31—C26	−174.58 (17)
C4—C5—C6—C1	1.0 (2)	C27—C26—C31—C30	−0.1 (3)
C2—C1—C6—C5	−0.3 (2)	C25—C26—C31—C30	−178.63 (18)
C2—C1—C6—C7	−179.44 (16)	C31—C30—C32—O5	114.9 (2)
C5—C6—C7—C8	−1.5 (2)	C29—C30—C32—O5	−60.3 (2)
C1—C6—C7—C8	177.65 (15)	C31—C30—C32—O6	−60.5 (2)
C6—C7—C8—C9	0.8 (2)	C29—C30—C32—O6	124.44 (19)
C6—C7—C8—C12	−170.38 (13)	C28—C29—C33—O4	152.52 (17)
C7—C8—C9—C10	0.3 (2)	C30—C29—C33—O4	−19.3 (2)
C12—C8—C9—C10	171.10 (14)	C28—C29—C33—C34'	−37.9 (5)
C7—C8—C9—C11	176.14 (14)	C30—C29—C33—C34'	150.3 (5)
C12—C8—C9—C11	−13.0 (2)	C28—C29—C33—C34	−19.9 (3)
C8—C9—C10—C5	−0.5 (2)	C30—C29—C33—C34	168.3 (2)
C11—C9—C10—C5	−176.55 (14)	O4—C33—C34—C39	107.5 (4)
C6—C5—C10—C9	−0.3 (2)	C34'—C33—C34—C39	9(3)
C4—C5—C10—C9	−179.18 (16)	C29—C33—C34—C39	−80.1 (5)
C10—C9—C11—O3	−42.1 (2)	O4—C33—C34—C35	−71.2 (5)
C8—C9—C11—O3	141.91 (16)	C34'—C33—C34—C35	−170 (3)
C10—C9—C11—O2	133.67 (17)	C29—C33—C34—C35	101.2 (5)
C8—C9—C11—O2	−42.3 (2)	C39—C34—C35—C36	−0.1 (7)
C7—C8—C12—O1	140.31 (16)	C33—C34—C35—C36	178.6 (3)
C9—C8—C12—O1	−30.5 (2)	C39—C34—C35—C41	−179.3 (5)
C7—C8—C12—C13	−35.8 (2)	C33—C34—C35—C41	−0.6 (8)
C9—C8—C12—C13	153.41 (14)	C34—C35—C36—C37	−1.3 (6)
O1—C12—C13—C14	115.45 (18)	C41—C35—C36—C37	177.9 (4)
C8—C12—C13—C14	−68.46 (18)	C35—C36—C37—C38	1.4 (5)
O1—C12—C13—C18	−62.0 (2)	C35—C36—C37—C42	−178.7 (4)
C8—C12—C13—C18	114.08 (16)	C36—C37—C38—C39	−0.1 (5)
C18—C13—C14—C15	1.5 (2)	C42—C37—C38—C39	179.9 (4)
C12—C13—C14—C15	−175.92 (13)	C37—C38—C39—C34	−1.2 (6)
C18—C13—C14—C20	178.44 (15)	C37—C38—C39—C40	−179.5 (4)
C12—C13—C14—C20	1.0 (2)	C35—C34—C39—C38	1.3 (7)
C13—C14—C15—C16	1.7 (2)	C33—C34—C39—C38	−177.4 (4)
C20—C14—C15—C16	−175.34 (15)	C35—C34—C39—C40	179.5 (4)
C14—C15—C16—C17	−3.5 (2)	C33—C34—C39—C40	0.8 (6)
C14—C15—C16—C21	175.84 (15)	O4—C33—C34'—C35'	−82.4 (12)
C15—C16—C17—C18	2.1 (2)	C29—C33—C34'—C35'	108.2 (11)
C21—C16—C17—C18	−177.21 (15)	C34—C33—C34'—C35'	8(2)
C16—C17—C18—C13	1.0 (2)	O4—C33—C34'—C39'	97.5 (13)
C16—C17—C18—C19	−176.44 (16)	C29—C33—C34'—C39'	−71.9 (13)
C14—C13—C18—C17	−2.8 (2)	C34—C33—C34'—C39'	−172 (4)
C12—C13—C18—C17	174.65 (14)	C33—C34'—C35'—C36'	−179.4 (9)
C14—C13—C18—C19	174.57 (16)	C39'—C34'—C35'—C36'	1(2)
C12—C13—C18—C19	−8.0 (2)	C33—C34'—C35'—C41'	0(2)
C27—C22—C23—C24	0.5 (3)	C39'—C34'—C35'—C41'	−179.7 (13)
C22—C23—C24—C25	0.3 (3)	C34'—C35'—C36'—C37'	−1.1 (17)
C23—C24—C25—C26	−0.5 (3)	C41'—C35'—C36'—C37'	179.3 (13)
C24—C25—C26—C27	−0.2 (3)	C35'—C36'—C37'—C38'	0(2)
C24—C25—C26—C31	178.26 (18)	C35'—C36'—C37'—C42'	179.2 (10)
C23—C22—C27—C26	−1.2 (3)	C36'—C37'—C38'—C39'	1(2)
C23—C22—C27—C28	−178.45 (18)	C42'—C37'—C38'—C39'	−178.2 (8)
C31—C26—C27—C22	−177.50 (17)	C37'—C38'—C39'—C34'	−1.0 (18)
C25—C26—C27—C22	1.0 (3)	C37'—C38'—C39'—C40'	−178.7 (12)
C31—C26—C27—C28	−0.1 (2)	C35'—C34'—C39'—C38'	0.3 (19)
C25—C26—C27—C28	178.40 (16)	C33—C34'—C39'—C38'	−179.6 (10)
C22—C27—C28—C29	177.19 (17)	C35'—C34'—C39'—C40'	178.1 (13)
C26—C27—C28—C29	−0.1 (3)	C33—C34'—C39'—C40'	−1.8 (18)

### Hydrogen-bond geometry (Å, °)

**Table:**

*Cg*1 and *Cg*2 are the centroids of the C22–C27 and C26–C31rings, respectively.

**Table:**

*D*—H···*A*	*D*—H	H···*A*	*D*···*A*	*D*—H···*A*
O2—H2A···O3^i^	0.82	1.81	2.6191 (17)	171
O6—H6A···O5^ii^	0.82	1.86	2.6709 (18)	170
C21—H21A···Cg7^iii^	0.96	2.85	3.475 (2)	124
C31—H31···Cg7	0.93	2.71	3.548 (2)	150
C42—H42B···Cg6^iv^	0.96	2.86	3.620 (6)	137

Symmetry codes: (i) −*x*+1, −*y*, −*z*; (ii) −*x*+1, −*y*+1, −*z*+1; (iii) −*x*+2, −*y*+1, −*z*+1;  (iv) *x*−1, *y*+1, *z*.
